# Effect of Phlorotannins from Brown Seaweeds on the In Vitro Digestibility of Pig Feed

**DOI:** 10.3390/ani10112193

**Published:** 2020-11-23

**Authors:** Lauren Ford, Chloe Curry, Mairead Campbell, Katerina Theodoridou, Gary Sheldrake, Jaimie Dick, Lorenzo Stella, Pamela J. Walsh

**Affiliations:** 1 School of Chemistry and Chemical Engineering, Queen’s University Belfast, Northern Ireland BT9 5AG, UK; l.ford@imperial.ac.uk (L.F.); chloe011358@hotmail.co.uk (C.C.); g.sheldrake@qub.ac.uk (G.S.); l.stella@qub.ac.uk (L.S.); 2 Institute of Global Food Security, Queen’s University Belfast, 19 Chlorine Gardens, Northern Ireland, Belfast BT9 5DL, UK; mcampbell105@qub.ac.uk (M.C.); k.theodoridou@qub.ac.uk (K.T.); j.dick@qub.ac.uk (J.D.); 3 School of Biological Sciences, Queen’s University Belfast, 19 Chlorine Gardens, Northern Ireland, Belfast BT9 5DL, UK; 4 Queen’s Marine Laboratory (QML) Queen’s University Belfast, 12-13 The Strand, Northern Ireland, Portaferry BT22 1PF, UK; 5 Atomistic Simulation Centre (ASC), School of Mathematics and Physics, Queen’s University Belfast, University Road, Belfast BT7 1NN, UK; 6 School of Mechanical Engineering, Queen’s University Belfast, The Asbhy Building, Stranmillis Road, Northern Ireland, Belfast BT9 5AJ, UK

**Keywords:** IVDMD, digestibility, brown seaweeds, pig nutrition, phenolics, phlorotannins, gut health

## Abstract

**Simple Summary:**

In large-scale pig farming, alternative and safe antimicrobials are needed to enhance pig nutrition. Seaweed bioactives, and in particular phlorotannins, have been reported to have antimicrobial properties. However, their effect on the digestibility of pig feed is not well understood. This study investigates the effect of these phenolics on the in vitro dry matter digestibility of seaweed using an in vitro pig digestibility model. The effect of the phenolics when extracted into their purified phlorotannin form, and blended directly into pig feed, was also tested using the same model. The results found that, when added to the pig feed, purified phlorotannins had a more pronounced effect on digestibility than seaweeds containing phenolics. In addition, the results showed that given the seasonal variation within seaweeds, inclusion of whole seaweeds should be based on phenolic concentration as opposed to percentage inclusion of seaweeds.

**Abstract:**

Phlorotannins have been reported to have positive effects on pig health, including improved gut health and digestibility. In this study, we investigate the effect of phenolics found in two brown seaweeds, *Ascophyllum nodosum* and *Fucus serratus*, on in vitro dry matter digestibility of seaweeds and commercial pig feed. Phlorotannin extracts and whole seaweeds were supplemented into pig feed to test their effect on digestibility. Solid-phase extraction was used to purify the phenolics to phlorotannins. The results showed a slight decrease in the digestibility of pig feed that was found to be significant when phlorotannin extracts were added from either seaweed. However, when whole *A. nodosum* was added to the pig feed, the effect on digestibility was less pronounced. Specifically, no significant difference in digestibility was observed at inclusion rates up to 5%, and thereafter results varied. A difference in digestibility was also observed in the same species at the same inclusion rate, collected from different seasons. This suggests that other compounds, e.g., polysaccharides, are having an effect on digestibility when whole seaweeds are supplemented to animal feed. This research has also highlighted the need to base supplementation on phenolic concentration as opposed to a standardised percentage inclusion of seaweeds to ensure that digestibility is not adversely affected.

## 1. Introduction

Due to the increasing human population across the world, the need to supply food products using more innovative and sustainable techniques has become of paramount importance. Edible seaweeds have been used widely in oriental cuisine for human consumption [1] and have shown in recent years that, if farmed or harvested sustainably, they are renewable [2,3]. The seaweed used in animal feeds commercially, however, has been limited to date. Seaweed as an animal feed supplement has been shown to enhance pig growth and help improve the digestibility in animal feeds by providing bioactive phytochemicals [4]. Seaweeds have also been used to feed livestock for thousands of years from the Ancient Greeks to modern-day Icelandic famers [5,6]. In Iceland, sheep, cattle and horses have all been fed processed seaweed, dried using geothermal heat, which reduces processing costs [7]. On the west coast of Scotland *Pelvetia*, a brown seaweed (*Phaeophyceae*) is fed to pigs in order to fatten them up before market [7]. Whilst there are some historical accounts of animal rearing using seaweed as a high proportion of the diet, there are fewer known commercial and scientific trials. A review by Corino et al. [8] summarises studies to date that have reported improvements in digestibility and overall animal health through the supplementation of seaweeds (and/or extracts) to animal feeds in pigs. In addition to their nutritional benefits, in recent years, seaweed compounds, specifically phenolics have gained significant attention for their potential antimicrobial [9,10,11] and anti-methanogenic [12,13] effects in livestock. Although pigs have the lowest methane emission when compared to other livestock, they still produce an estimated CH_4_ emission of 0.8, 2.4 and 8.2 g/head for weaned piglets, fattened pigs and sows, respectively [14].

Seaweed is an advantageous aquatic plant to be incorporated into feeds from a sustainability aspect as it does not compete with land space for food crops and does not require fertiliser in order to grow [15,16]. Seaweed can also be sustainably farmed or harvested from naturally growing sources, allowing natural regeneration [15]. However, seaweed incorporation into animal diets, especially for monogastric animals such as pigs, has had largely varied results. An early study (1979) by Jones et al. [17] reported that the supplementation of brown seaweed into pig diets at high inclusion rates of 10% has a negative effect on weight gain. A study by Michiels et al. [18], however, showed weight gain after 11 days post weaning when compared to the control diet when the pig feed was supplemented with between 2.5 and 10 g/kg of *A. nodosum* (whole feedstock). Inclusion of sun-dried *A. nodosum* in the diet at 20 g/kg for 7 days has also been shown to reduce faecal shedding of *E. coli* 0157:H7 in cattle [19]. Other studies have used seaweed extracts that contain the polysaccharides laminarin and fucoidan. The inclusion of these extracts allowed for a lower lactose content in the feed given to post weaning piglets and was found not to adversely affect the growth rate [20]. McDonnell et al. [21] reported that by supplementing the diet of weaning piglets with laminarin- and fucoidan-containing seaweed species, they also showed an increased daily weight gain. Most digestibility studies, however, do not access the impact of seasonal variation, which is important for commercial application. In addition, there is a lack of understanding of the effect of different seaweed compounds on dry matter digestibility, particularly in the case of phenolics.

Seaweeds are grouped into three phyla according to their pigmentation, known commonly by colour: Chlorophyta (green algae), Phaeophyta (brown algae) and Rhodophyta (red algae) [22]. Each hosts unique phytochemicals (e.g., lipids, phenolics, and alkaloids) and complex carbohydrates, mainly in the form of sulphated (e.g., carrageenan) and non-sulphated (e.g., alginate) polysaccharides. The carbohydrate content in all seaweeds is high. However, brown seaweeds tend to have the highest non-starch polysaccharide content, whereas red and green seaweeds have higher amino acid content [23]. Livestock diets do not typically contain these complex polysaccharides and literature studies to date of their digestibility in both ruminant and monogastric animals are limited. However, some brown seaweeds, including *Laminaria digitata*, *A. nodosum* and *Fucus vesiculosus*, have also been used commercially in animal feeds [24], suggesting that their dietary properties are comparable to terrestrial-based fed ingredients. One commercial seaweed feed, *OceanFeed Swine^®^*, which is a blend of different seaweed species, has been reported to have positive results in terms of daily weight gain and feed efficiency in nursery and pig-fattening stages of development [25].

Seaweeds are a largely seasonal plant containing varied amounts of phenolics, fatty acids, protein and minerals throughout the year [26,27,28,29]. Brown seaweeds contain phenolic compounds known as phlorotannins, which are unique to this class of seaweed. Although these phenolic compounds are commonly compared to the terrestrial tannins, they are structurally very different [5]. The phlorotannins in seaweeds, however, do have some effects similar to terrestrial plant tannins such as binding to protein [30,31]. Gulzari et al. [32] hypothesised that phlorotannins from seaweed-supplemented soybean meal formed complexes with proteins and fibres in the meal and reduced its overall digestibility in the rumen. However, few studies have looked specifically at the effect of phlorotannins on the digestibility of meal in ruminant or monogastric animals.

The aim of this study was to test the in vitro dry matter digestibility of the brown seaweeds that were rich in phenolics in order to assess the correlations between their digestibility and the phlorotannins levels. Previous studies have shown that phlorotannins could potentially help improve the gut health of pigs [8,10]. However, a review by Huang et al. [33]. highlighted that terrestrial tannins can inhibit digestion when added at high concentrations in livestock diets. Therefore, it is important to understand the effect of phlorotannins on digestibility in order to ensure that their addition does not negatively impact on the weight gain of the animals. In addition, whilst phlorotannins act in a similar way to condensed and hydrolysed tannins from terrestrial plants, they are structurally very different, and little is currently known about their effects in the digestive tract of livestock, in particular pigs. This study investigates the dry matter digestibility of two brown seaweeds, *A. nodosum* and *F. serratus*, that are known to be rich in phenolics and the effect of phenolic content on the digestibility of the whole seaweed biomass. The phenolic content in brown seaweeds is known to vary seasonally [10,28], and therefore seaweed collected from different seasons were used to study this effect. To better understand the effect of phenolics on the digestibility of the seaweed biomass, a further in vitro study was performed with and without polyethylene glycol (PEG). Some methane migration studies have shown that PEG inhibits the activity of phenolics [13,34]. This approach was therefore taken to help better understand the role of phenolics in seaweed biomass digestibility. Phenolic compounds and tannins are known to bind to proteins and fibre, with a higher affinity for the protein fractions [35]. Therefore, purified phlorotannin extracts were isolated from the phenolic fraction and blended with protein-rich commercial pig feed to assess the specific effect of the phlorotannins as opposed to the whole seaweed that contains other macromolecules including chlorophylls, carotenoids and complex mono-/polysaccharides that might affect digestibility.

## 2. Materials and Methods

### 2.1. Collection of Seaweed Samples

*A. nodosum* and *F. serratus*, two intertidal brown seaweeds, were collected by hand at the end of each month from an artificial lagoon behind a breakwater in Bangor, Northern Ireland (54°39′58.6′′ N 5°39′53.4′′ W). Samples were identified by a marine phycologist, collected and stored in plastic sample bags to be transported to the laboratory. Within 3 h of collection, the samples were washed with fresh water to remove particulates and grazing species before being frozen for storage at −20 °C. The whole plant was used for each sample collected. Approximately 200 g wet weight was collected for each species at each collection point. The dry matter was the mass of sample after freeze drying taken away from the mass of the sample before freeze drying.

### 2.2. Seaweed Processing

Frozen seaweed samples were lyophilised, then ground using a Polymix PX-MFC 90D mechanical grinder fitted with a 2 mm sieve. Samples were weighed before and after lyophilisation to determine the dry matter (DM) of samples. They were then placed in plastic bags and stored in the freezer at −20 °C until used for analysis. Samples for commercial feed study were purified to ensure that the activity measured was due to the phlorotannins only. Samples were purified by solid-phase extraction (SPE) using Amberlyst XAD 7HD. Dried seaweed (approximately 10 g) was stirred using a magnetic flee with acetone/water (7:3 *v*/*v*, 200 mL) for 3 h at room temperature. The seaweed extracts were then centrifuged (5000 rpm for 10 min). The supernatant was placed on the rotary evaporator to remove the acetone. The aqueous mixture was then added to the Amberlyst XAD 7HD beads (10 g) in a solid-phase extraction (SPE) column. The column was first eluted with water (3 × 200 mL) and then with ethanol (3 × 100 mL). The ethanol was then removed by rotary evaporation and then the dried material was stored in the freezer until it could be used for biological analysis.

### 2.3. Proximate Analysis

#### 2.3.1. Proximate Measurements

The ash content, acid detergent fibre (ADF) and neutral detergent fibre (NDF) were measured using the same methods as reported by Van Soet et al. [36]. Briefly, ground seaweeds were analysed for NDF by boiling in neutral detergent (ND) solution, amylase and sodium sulphite for 1 h. For ADF, the ground seaweed was boiled for 1 h in acid detergent solution. Crude protein (CP) was calculated by measuring the nitrogen content and multiplying it by 5 [37]. The nitrogen content was measured using the Dumas method using Leco Protein/N Analyser (FP-528, Leco Corp., St Joseph, MI, USA) [38]. Gross energy (GE) content was determine by AFBI Hillsborough Analytical Services using an isothermal automated bomb calorimeter (PARR Instrument, Model 6300, Illinois, IL, USA).

#### 2.3.2. In Vitro Dry Matter Digestibility (IVDMD)

In vitro digestion was measured using a technique adapted from Tiwari and Jha [39]. A three-step enzymatic digestion technique was used to determine the apparent total tract digestibility of dry matter (DM). An amount of 1 g of digestible sample was placed in a flask. Then 50 mL of phosphate buffer solution 1 (0.1 mol/L, pH 6.0) was added to the flask followed by 20 mL of hydrochloric acid (HCl) solution (0.2 mol/L). The pH was adjusted to 2.0 by mixing with HCl solution (1.0 mol/L) or sodium hydroxide (NaOH) (1.0 mol/L). A volume of 1 mL of chloramphenicol (34 mg/mL in ethanol) was added to the solution to prevent bacterial growth during hydrolysis. A volume of 2 mL of freshly prepared pepsin solution was added to the solution which was prepared by adding pepsin (0.75 g, Sigma CAT NO 9001-75-6, Dorset, UK) to 30 mL of ultra-pure water. The flask was then closed and incubated in a water bath at 39 °C for 2 h. A volume of 20 mL phosphate buffer solution 2 (0.2 mol/L, pH 6.8) was then added to the samples and 10 mL of NaOH solution (0.6 mol/L). The pH was adjusted to 6.8 with HCl (1 mol/L) or NaOH (1 mol/L) and 6 mL of pancreatin solution was added. Pancreatin solution was made by adding 3 g pancreatin (Sigma CAT NO 8049-47-6) to 90 mL ultra-pure water. Hydrolysis was then continued under the same conditions for a further 4 h.

The IVDMD was calculated as follows:

IVDMD = (DWH − DWR)/DWH × 100

where

DWH = dry weight of sample before hydrolysis;

DWR = dry weight of residue.

To counteract the effect of the phlorotannins on digestibility, a solution of PEG (MW 4000) (Fluka Research Chemicals) in water was added in different volumes to achieve the required PEG: phlorotannin (PT) ratio. Phlorotannins were extracted from seaweeds collected in May as this month had the highest concentration of phlorotannins and required less biomass to obtain the concentration required. A stock solution of PEG 4000 was prepared at a concentration of 5 mg/mL PEG in water. The stock solution was sonicated until fully solubilised. The concentration of the phenolic content of seaweeds were calculated using the Folin–Ciocalteu (FC) assay which gives the concentration of phenolics in phloroglucinol equivalents mg/g of seaweed (see Section 2.4). The volume required of PEG stock solution was then calculated for each seaweed digestibility based on weight/weight calculations. Molar equivalents are not possible when using biological matrixes such as these due to the unknown molecular mass of the mixture of compounds present in the extracts.

### 2.4. Folin–Ciocalteu (FC) Assay

The phenolic content was measured using the FC assay using a method described by Ford et al. [10]. Briefly, phenolic extracts were prepared as per Section 2.2 (seaweed processing). The resultant extract was then diluted ×40 before it was added to the FC reagent at a dilution of 1:1 with deionised water (0.5 mL). After 5 min, 20% aqueous sodium carbonate was added (2.5 mL). The solution was then left to stand for 40 min in the dark before the absorbance was recorded at wavelength of 755 nm on a Jenway 6305 fixed-wavelength spectrophotometer. The absorbances were converted to a concentration in phloroglucinol equivalents using a calibration curve (*R^2^* > 0.95) of phloroglucinol.

### 2.5. Gel Electrophoresis

Samples were prepared using seaweed polyphenols previously extracted and purified using solid-phase extraction and produced a water-soluble powder. Standard samples were prepared at a concentration of 1 mg/mL in deionised water of the phlorotannins (extracted from seaweeds collected in May) and pepsin enzyme (Sigma) and PEG (2000 and 4000 MW). When mixtures were used, samples were prepared at 1:1 ratio (w/w). After preparation, the samples were centrifuged for 5 min at 5000 RPM to remove particulates. Sample solution (30 µL) was added to LDS buffer (10 µL, Invitrogen) and vortexed. This solution was then added to 10 well gel plates (NuPAGE 4–12% BisTris gels) along with a PageRuler pre-stained protein ladder (Thermo Scientific, Loughborough, UK) which was run in NuPAGE MES SDS running buffer at 200 V for 30 min. A Powerease 300 W powerpack was used with a Xcell sure-lock gel chamber for running the gel plates. After 30 min, the gel plates were removed from the sure-lock chamber and carefully removed from the plastic casing. The gel was then placed in staining solution (Coomassie^®^ brilliant blue; 0.25 g in 125 mL ethanol, 100 mL deionised water and 25 mL acetic acid) for 4 h. After 4 h, the gel was removed and left overnight in de-staining solution 1:1:8 *v*/*v* methanol:acetic acid:water). The gel was then photographed.

### 2.6. Statistical Analysis

**Figure 1:**
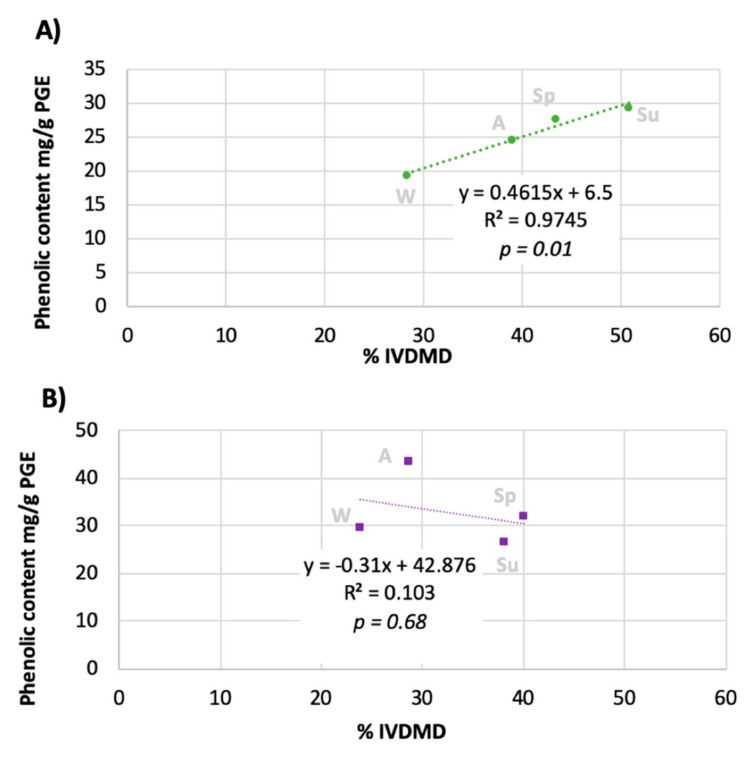
Correlation of phenolic content in phloroglucinol equivalents mg/g (DM) to in vitro digestibility of dry matter (%IVDMD) for *A. nodosum* (**A**) and *F. serratus* (**B**).

All the ANOVA statistical calculations were performed using IMB SPSS v25 (SPSS Inc, Chicago, IL, USA). All data sets were analysed using one-way ANOVA and post hoc with LSD multiple comparison test, with the exception of Figure 1. For this data set, linear regression analysis was performed with a significant test for regression on the data in excel to test the null hypothesis that phlorotannin content will have no effect on digestibility. A significance level of *p* < 0.05 was used for statistical analysis.

## 3. Results

### 3.1. Proximate Analysis

The in vitro dry matter digestibility of the brown seaweeds was studied to determine the effect of phenolics in a monogastric model. The proximate analysis investigated included GE, CP, ADF, NDF, DM, ash, IVDMD and phenolic content. The results of this analysis are shown in Table 1. In terms of CP, there was no significant difference found between *A. nodosum* and *F. serratus*, throughout the year, with the exception of *F. serratus* in spring, which showed a significant (*p* < 0.001) increase in CP from 14.5 ± 1.2 to 20.7 ± 1.2%. Overall, the CP was found to be significantly higher (*p* < 0.05) in winter and spring for both species.

The NDF and ADF measurements are given in Table 1. The effect of seasonal variation was apparent in *A. nodosum* in ADF data, with an exponential increase from summer to winter from 28.4 ± 5.8 to 48.9 ± 0.8%, although the increase was only found to be statistically significant between summer and autumn (*p* = 0.002), whereas no seasonal effect was observed in the ADF results for *F. serratus*. No statistically significant difference was found in the NDF results for *F. serratus* between seasons.

When comparing the phenolic content between seaweed species (Table 1), no significant difference was observed between the phenolic content of *A. nodosum* when compared to *F. serratus* in spring and summer (*p* = 0.120), but there was a significant difference (*p* < 0.05) between species in both autumn and winter. In autumn, *F. serratus* showed the greatest increase in phenolic content to 43.4 ± 2.6 (*p* < 0.001) mg/g compared to compared to 29.4 ± 1.4 (*p* < 0.001) for *A. nodosum*. To better understand the relationship between the phenolic content and digestibility, the phenolic content was correlated to the IVDMD, as shown in Figure 1. A linear response was observed for the relationship between phlorotannin content and IVDMD for *A. nodosum* with R^2^ value 0.9745 (Figure 1A), which was found to be statistically significant (*p* = 0.01). In *F. serratus*, the relationship between phenolics and IVDMD showed the opposite trend, whereby a negative correlation was observed. This, however, was not statistically significant for IVDMD (Figure 1B).

### 3.2. Effect of Phenolics on Digestibility with and without PEG

**Figure 2:**
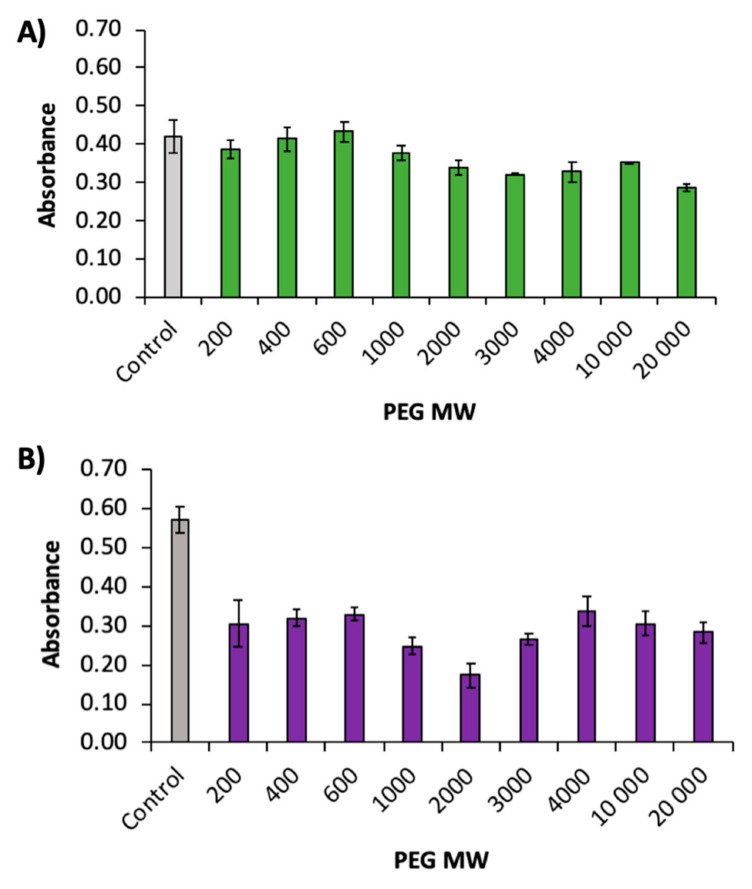
Change in absorbance of the FC assay upon binding to different PEG MW for purified phlorotannins from (**A**) *A. nodosum* and (**B**) *F. serratus* extracted from samples collected in May.

Purified phlorotannin extracts were complexed with different molecular weights (MW) of PEG to test which would bind the best to the phlorotannins in *A. nodosum* and *F. serratus* (Figure 2). The complexes were tested with the FC assay.

In *A. nodosum*, there was no significant difference between the control and PEG of a MW of 200 to 600. However, for PEG 1000 MW upwards, a statistically significant decrease (*p* < 0.005) in absorbance was observed. However, in *F. serratus*, all molecular weights were found to have a significant decrease in absorbance (*p* < 0.001) with respect to the control.

**Figure 3:**
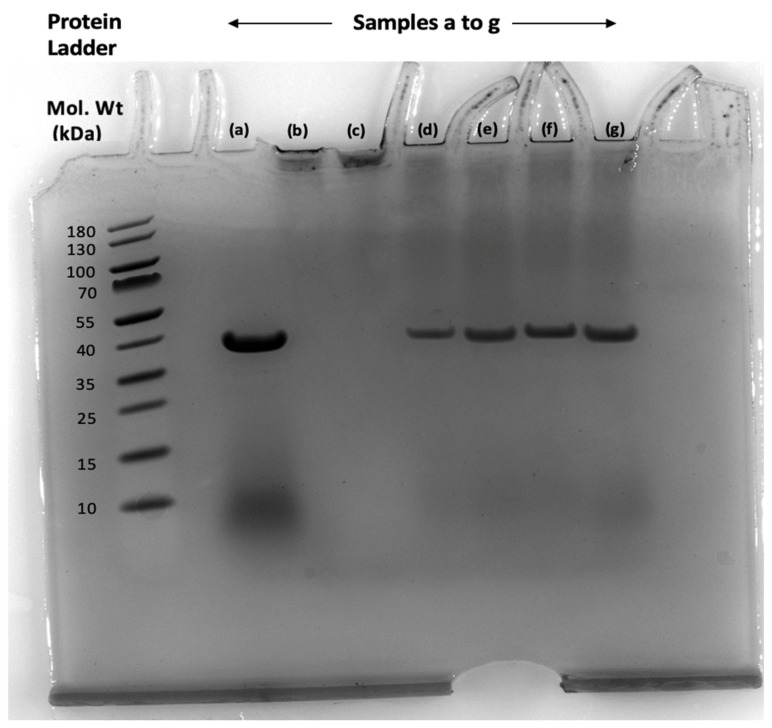
Gel electrophoresis of pepsin with and without binding to the tannins and PEG. From left to right after protein ladder: (**a**) pure pepsin, (**b**) pepsin + Asco phlorotannin, (**c**) pepsin + Fucus phlorotannin, (**d**) pepsin + Asco phlorotannin + PEG 2000MW, (**e**) pepsin + Asco phlorotannin + 4000MW, (**f**) pepsin + Fucus phlorotannin + PEG 2000MW and (**g**) pepsin + Fucus phlorotannin + PEG 4000MW.

To verify that PEG was inhibiting the effect of the phlorotannins, gel electrophoresis was used to measure the change in MW of proteins when using pepsin, which was used as an enzyme in the in vitro study. Pepsin was bound to purified phlorotannin and showed a distinct change in the MW when compared to the protein ladder. When PEG was incorporated into the matrix, the spot for the phlorotannin–protein complex was diminished and the pure pepsin spot was recovered (Figure 3). The results suggest that the phlorotannins are binding to the enzymes in the digestive tract but the enzyme was recovered when PEG is added, therefore confirming that PEG is counteracting the effect of the phlorotannins on the proteins.

Digestibility experiments were set up with and without PEG to measure the effect that the phlorotannins are having on the digestibility of the two seaweed species over the year. Given that gel electrophoresis showed no difference, 4000 MW was selected for the following studies. PEG was added into the seaweed at ratios of 1:1, 1:3 and 1:5 phlorotannin: PEG, the phlorotannin content was calculated using the FC assay and expressed in mg/g PGE.

**Figure 4:**
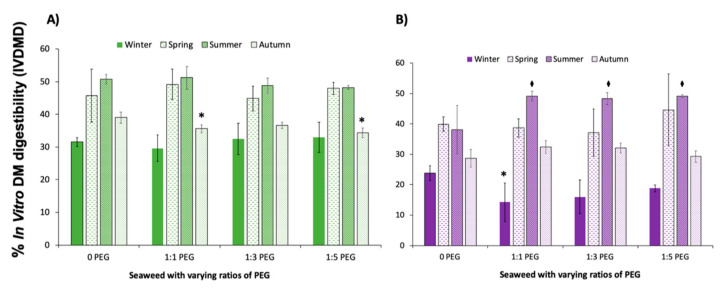
In vitro DM digestibility (IVDMD) profiles of (**A**) *A. nodosum* and (**B**) *F. serratus* with and without PEG. Three different ratios of PEG were used: 1:1, 1:3 and 1:5 phlorotannin:PEG (*p* < 0.05, * = significant decrease; ♦ = significant increase).

Figure 4 shows the effect on digestibility when PEG 4000 MW is added to the seaweed samples in the in vitro digestibility study. There was no significant decrease in the IVDMD when PEG was added at any ratio, in any season accept for winter for *A. nodosum*. In autumn, however, there was a statistically significant decrease in IVDMD when PEG was added at a 1:1 ratio (*p* = 0.015) and 1:5 ratio (*p* = 0.003) when compared to the PEG 0 control. In *F. serratus*, a significant decrease was observed in winter at a 1:1 ratio (*p* = 0.028), whereas there was a significant increase in digestibility in summer at all ratios: 1:1 (*p* = 0.012), 1:3 (*p* = 0.017), 1:5 (*p* = 0.011). The phenolic content (Table 1) in *F. serratus* was found to be the lowest (*p* < 0.05) at 26.49 ± 1.42 mg/g in summer compared to the other seasons tested in this species.

Given the mixed results in this study, purified extracts were isolated to study the specific effect of the phlorotannins on the digestibility, which is discussed in the next section.

### 3.3. Effect of Phlorotannins on Commercial Pig Feed

**Figure 5:**
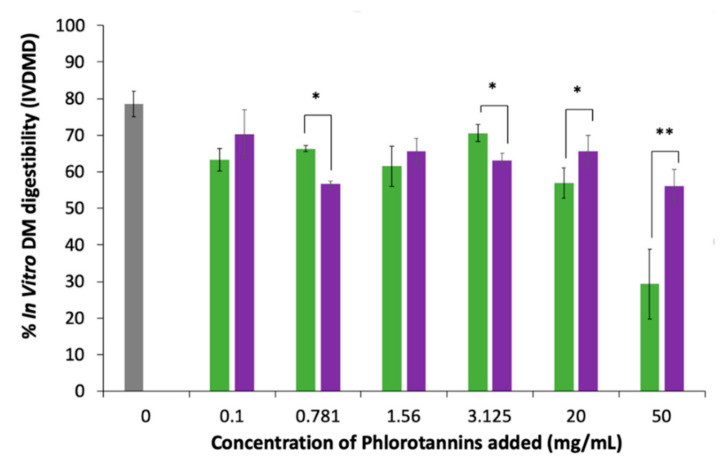
In vitro DM digestibility (IVDMD) of a commercial pig feed when different concentrations of phlorotannins isolated from *A. nodosum* (purple) and *F. serratus* (green) in May were added. Control (commercial pig feed, grey) (* = *p* < 0.05; ** = *p* < 0.001).

Purified phlorotannins extracts were mixed at different concentrations with a commercial feed and their effect on digestibility was recorded. The results are shown in Figure 5. A significant decrease (*p* < 0.05) in IVDMD was observed in *A. nodosum* (Figure 5) between the control and all concentrations of phlorotannins added. The same trend was also observed in *F. serratus*. However, when comparing *A. nodosum* to *F. serratus*, there was an observed difference in digestibility between both species when phlorotannins were added. This was found to be statistically significant at concentrations of 0.781, 3.125, 20 and 50 mg/mL. However, at lower concentrations of 0.781 and 3.125 mg/mL, IVDMD was found to be higher in *A. nodosum* than *F. serratus*, whereas the opposite trend was observed at higher concentrations of 20 and 50 mg/mL. When pure phlorotannin extracts were added directly to the pig feed, the results clearly show that phlorotannins found in both *A. nodosum* and *F. serratus* affect the digestibility of the pig feed. At lower concentrations of <3.125 mg/mL, *F. serratus* was less detrimental to the digestibility of a high protein feed than *A. nodosum*, whereas the opposite occurs above 3.125 mg/mL.

### 3.4. Effect of Seaweeds Added to Commercial Pig Feed

**Figure 6:**
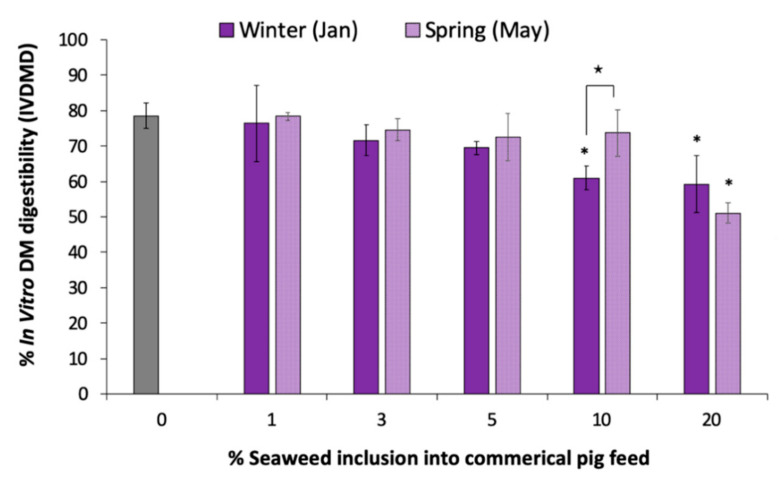
Change in in vitro DM digestibility (IVDMD) of a commercial pig feed when different percentages of *A. nodosum* were added from samples collected in Jan and May. * = *p* < 0.05 between control and % inclusion; ⋆ = *p* < 0.05 between pairs.

Finally, the same digestibility study was repeated using the whole seaweed plant (without PEG), as opposed to just the phlorotannin extracts, in order to investigate the effects of other compounds found in *A. nodosum* on the digestibility of the pig feed, in addition to the phlorotannins. Our results (Figure 6) show that there was no significant difference in digestibility between samples collected in winter and spring at up to 5% inclusion, which is equivalent to 0.95 and 1.9 mg/g (Table 2) of phlorotannins, respectively, in comparison to the control (pig feed only). At 10% inclusion, a significant drop (*p* < 0.01) in digestibility was observed from 77.8 (±3.5) % to 61.0 (±3.3) % in winter (TPC ≈1.9 mg/g), whereas no significant difference in digestibility was observed in May, which had a significantly higher phenolic content (TPC ≈3.8 mg/g). At 20% inclusion, the opposite trend was observed, whereby the higher concentration of phenolics, ≈7.6 mg/g in May showed a lower digestibility of 51.0 (±2.8) %, compared to winter which was 59.2 (±8.1) %. However, this was not found to be significant.

## 4. Discussion

Brown seaweeds are relatively low in protein compared to other feed sources, such as alfalfa or soy [40,41]. The highest CP contain in this study was found in winter and spring for both species and is likely caused by N-based fertilisers leaching into coastal areas, as terrestrial crops and grasslands tend to be fertilised during this period [42]. Al-Yaman et al. [43] reported that the phenolic contents in brown seaweeds correlate to their protein content. However, no correlation was observed between CP and phenolics in this study. It is noteworthy that the species of brown seaweeds differed between these two studies.

The NDF results of the seaweeds studied have been found to be comparable to other terrestrial forage, e.g., alfalfa [44,45], corn and wheat [46], which have been reported to be approximately 45%. In the ADF results, no seasonality effect was observed in either seaweed species. The difference was not found to be statistically significant between seasons. Typically, ADF <35% are desirable in livestock diets [47]. In this study, the digestibility of the whole seaweed of *F. serratus* was found to have lower digestibility than *A. nodosum* seaweed, with ADF in the range of 21 to 26% and 15 to 18%, respectively. While a correlation between phenolic and ADF content for *A. nodosum* was observed, this trend was not found in *F. serratus*. The polymer content might also account for the difference in digestibility. In brown seaweeds, the polymers are mainly alginates and sulphated fucoidans [48,49]. In humans, alginate has been reported to be indigestible and is likely to have the same effect in pigs given that our digestive systems are very similar [50]. It is considered beneficial as an anti-obesity fibre that promotes weight loss [51,52], whereas fucoidan has been reported to have no effect on weight loss in humans in a recent obesity study [53]. Therefore, it is likely than fucoidan is more digestible than alginate in brown seaweeds. Fletcher et al. [54] have reported the fucoidan content in *A. nodosum* is significantly higher than *F. serratus* throughout the year Therefore, a higher fucoidan content in *A. nodosum* may have contributed to the significant difference in ADF.

When comparing IVDMD to total phenolic content, the different trend observed between *A. nodosum* and *F. serratus* suggests that seaweed species produce an effect on digestibility. Studies of phenolic compounds in seaweeds have reported that phlorotannin structures vary between species [55,56,57]. It is likely that phlorotannins with different linkages, functional groups, reactivities and molecular weights interacted and/or bound differently with other macromolecules within the seaweeds, namely polysaccharides and proteins, thus causing a different effect on digestibility.

Polyethylene glycol (PEG) is used to counteract the effect of tannins in the animal feed [6]. This method has been used for terrestrial tannins and with phlorotannins from brown seaweed [35]. Polyvinylpyrrolidone (PVP) and PEG have been used to bind to tannins for the last three decades. However, the same has been performed on conventional terrestrial tannins [6]. The use of PEG and PVP with molecular weights (MW) between 2000 and 35,000 has been tested for their binding abilities with terrestrial tannins [58], with the most commonly used molecular weights between 3500 and 4000 [6]. The FC assay is a colorimetric assay based on the redox potential of the substrate and its ability to complex with the metallic FC reagent [59]. The reaction produces a dark blue colour upon complexation and this can be measured at 755 nm. Therefore, the PEG MW best at counteracting the effect of the phlorotannins should have the largest decrease in absorbance. In the PEG binding study, the most pronounced decrease was observed in *F. serratus* at PEG 2000 MW. This would suggest that PEG 2000 was the best phlorotannin binder for *F. serratus* phlorotannins. However, it is noteworthy that there was an obvious precipitate formed from 1000 to 20,000 MW in both seaweeds tested, which could have caused higher readings in the absorbance due to turbidity and the precipitates absorbing the light at all wavelengths. Further, the FC assay is a metal complex colorimetric reaction and therefore the affinity between the phlorotannins and FC reagent is likely to be very different from that of the affinity between the tannin and protein.

The gel electrophoresis showed that both PEG 2000 and 4000 MW successfully inhibited phlorotannins. Previous studies have reported PEG 3350 MW at blocking phlorotannin [34] and most terrestrial tannins studies have reported between 3500 and 4000 MW to be most effective [6]. Therefore, 4000 MW was used to mask the effect of phenolics in the subsequent study IVDMD study. As PEG counteracts the effect of the phlorotannins, the results would suggest that PEG was only successful at blocking the phlorotannins in *F. serratus* in summer at all ratios. However, at higher phenolic concentrations, no significant difference in digestibility was observed, suggesting that the ratios and ME of PEG used have not been successful at blocking the phlorotannins. The same trend was observed in *A. nodosum*, whereby there was no significant difference in IVDMD between the control and the samples complexed with PEG, suggesting that the PEG ratio and MW used was not sufficient to bind this concentration of phenolics. PEG may have been less effective in the IVDMD study compared to binding study (Figure 2) and gel electrophoresis (Figure 3) due to pH. The effect of pH on the binding efficiency of terrestrial tannins has previously been reported by Makkar et al. [58].

The results of the PEG binding digestibility study (Figure 4) suggest that the phenolics found in *F. serratus* in summer do affect digestibility, whereas no relationship was found when correlating the IVDMD to phenolics (Figure 1). The lack of correlation in the results shown in Figure 1 may be a result of the limitations in the FC assay, as discussed in a recent review by Ford et al. [60], as opposed to a lack of relationship between phenolics and digestibility, thus highlighting the need to improve characterisation methods used to measure phenolics in seaweeds. Further, as discussed above, pH may have affected the correlation. In addition, these studies have investigated the digestibility of the whole seaweed including the polymer fraction and other compounds as previously discussed, as opposed to the specific relationship between the phlorotannins on the animal feed used. The most common method of inclusion to animal feeds is the inclusion (between 1 and 5%) of the whole seaweed feedstock [5], as opposed to the crude (phenolics) or purified (phlorotannins) due to animal feed regulations governing the use of extracts.

The results (Figure 5) clearly show that phlorotannins can negatively affect digestibility of the pig feed. However, when whole seaweed (Figure 6) was added, the effect was significantly reduced. This would suggest that the other compounds (most likely the polysaccharides) which form the seaweed matrix are inhibiting the interaction between the phlorotannins and digestive enzymes, thus counteracting the reduction in digestion. The results also show that higher concentrations of phenolics can be added by using the whole feedstock as opposite to purified phlorotannin extracts before effecting digestibility. However, in spring, 10% seaweed inclusion with ≈3.8 mg/g did not affect digestibility, whereas 20% seaweed inclusion with ≈3.8 mg/g in winter significantly reduced digestibility, thus confirming that other compounds, or a variation in phenolic structure, may be affecting digestibility. In the literature, the most common inclusion rates reported are between 1 and 5% [5,30,41]. Although this would suggest that supplementing whole seaweeds, as opposed to phlorotannin extracts, might be better to counteract the effect of phlorotannins on digestibility, it is noteworthy to consider that the acetone/water yields may extract higher concentrations of phlorotannins from the biomass than the buffer system used in the digestibility studies, thus also masking the effect of the phlorotannins on digestibility. Further work is required to better understand the digestibility of the polysaccharides, types of phlorotannins and other compounds found in seaweeds to determine the overall effect of adding seaweeds to pig feed.

## 5. Conclusions

The work herein shows a clear correlation between the digestibility of pig feed and phenolics, when the phenolics were purified in phlorotannin. In the two seaweeds studied, *A. nodosum* and *F. serratus*, the addition of phlorotannin extracts caused a significant decrease in IVDMD. This relationship becomes more complex when the whole seaweed plant was added. Further work is required to better understand the inclusion of whole seaweeds in feeds. In addition, consideration needs to be given to the effect of seasonality on the chemical composition of seaweed being added, as phenolics can vary significantly within the same species during the year. Ideally, the seaweed should be harvested when the phenolics are most abundant in order to reduce the overall amount of seaweed that needs to be supplemented. This research has highlighted that supplementation should not just be based on standardised percentage inclusion of seaweeds, but on the chemistry of biomass (e.g., phenolic and biopolymers) to mitigate the effect on digestibility. To validate the use of seaweeds in pig feed, an animal trial is required to gain a better understanding of the in vivo digestibility of seaweed-supplemented pig feeds before seaweeds could be considered for use in commercial pig feed. In addition, further work is required on the digestibility of other seaweed compounds and the bioavailability of the pholorotannins to the animal if whole seaweed is to be supplemented.

## Figures and Tables

**Table 1:** Proximate analysis of *A. nodosum* and *F. serratus*. All parameters are expressed in terms of % of the overall biomass unless otherwise stated.

Species of Seaweed	Season	Gross Energy MJ/kg DM	Dry Matter		Ash		Crude Protein (CP)		Acid Detergent Fibre (ADF)		Neutral Detergent Fibre (NDF)		IVDMD		Phenolic Content FC Assay mg/g (DW)	
			Ave	SD	Ave	SD	Ave	SD	Ave	SD	Ave	SD	Ave	SD	Ave	SD
*A. nodosum*	Winter (Jan)	13.92	89.23	0.45	30.20	2.45	11.58	3.06	16.90	0.58	48.48	0.82	28.24	6.78	19.20	2.74
	Spring (April)	14.02	88.50	0.05	28.36	0.38	14.50	1.19	15.34	4.68	40.26	3.29	43.32	1.26	27.46	2.95
	Summer (July)	15.08	88.66	0.20	22.61	1.23	7.27	2.22	15.04	1.68	28.39	5.80	50.79	1.47	29.28	1.26
	Autumn (Nov)	14.61	88.38	0.06	23.49	0.39	6.79	1.34	18.79	0.87	41.63	1.32	39.01	2.41	24.53	1.39
*F. serratus*	Winter (Jan)	14.39	89.70	0.06	33.15	0.39	13.29	1.34	21.94	0.87	42.83	1.32	23.85	2.41	29.42	1.39
	Spring (April)	15.16	90.78	0.60	26.94	3.37	20.73	1.53	24.54	3.51	45.04	7.73	39.99	2.42	31.70	2.96
	Summer (July)	16.55	89.83	0.53	19.34	0.66	7.77	0.69	22.81	1.82	43.73	4.69	38.10	7.93	26.49	1.42
	Autumn (Nov)	15.06	89.50	0.07	26.44	1.28	9.85	1.08	25.71	2.15	44.49	5.21	28.69	2.88	43.40	2.56

**Table 2:** Concentration of phlorotannins at each inclusion rate.

% Inclusion		1	3	5	10	20
TPC (mg/g) DW	Winter	0.19	0.57	0.95	1.9	3.8
	Spring	0.38	1.14	1.9	3.8	7.6
